# Dietary Adherence and Satisfaction with a Bean-Based High-Fiber Weight Loss Diet: A Pilot Study

**DOI:** 10.1155/2013/915415

**Published:** 2013-10-29

**Authors:** Tonya F. Turner, Laura M. Nance, William D. Strickland, Robert J. Malcolm, Susan Pechon, Patrick M. O'Neil

**Affiliations:** Department of Psychiatry and Behavioral Sciences, Medical University of South Carolina, 67 President Street, Suite 410, Charleston, SC 29425, USA

## Abstract

*Objective*. Dietary fiber can reduce hunger and enhance satiety, but fiber intake during hypocaloric weight loss diets typically falls short of recommended levels. We examined the nutritional effects and acceptability of two high-fiber hypocaloric diets differing in sources of fiber: (a) beans or (b) fruits, vegetables, and whole grains. *Methods*. Subjects were 2 men, 18 women, mean age = 46.9, and mean BMI = 30.6. Subjects completed 3-day food diaries in each of the two baseline weeks. Subjects were then randomized to four weeks on one of two 1400-calorie diets including 25–35 g fiber primarily from 1.5 cups beans/day or from fruits, vegetables, and whole grains. Recommended fiber-rich foods were provided. Subjects kept weekly 3-day food diaries and were assessed weekly. *Results*. Diet conditions did not differ on outcome measures. Both diets increased fiber intake from 16.6 g/day (SD = 7.1) at baseline to (treatment average) 28.4 g/day (SD = 6.5) (*P* < 0.001). Fiber intake was consistent over treatment. Caloric intake dropped from 1623.1 kcal/day (SD = 466.9) (baseline) to 1322.2 kcal/day (SD = 275.8) (*P* = 0.004). Mean weight loss was 1.4 kg (SD = 1.5; *P* < 0.001). Energy density and self-reported hunger decreased (*P*'s < 0.01) while self-reported fullness increased (*P* < 0.05). Both diets were rated as potentially acceptable as long as six months. *Conclusions*. Both diets significantly increased fiber intake by 75%, increased satiation, and reduced hunger. Results support increasing fiber in weight loss diets with a variety of fiber sources.

## 1. Introduction

The rising prevalence and disease burden of obesity are well documented. Modest weight loss (5–10%) helps to reduce the risk of developing many of comorbidities of obesity, potentially decreases their severity, and has been associated with increased mood and quality of life.

Numerous studies have examined the effects of variations in the components of hypocaloric diets used for weight loss, primarily macronutrient composition. The effects of fiber level have received less attention than that given to macronutrient composition. Epidemiologic data show that fiber intake is inversely associated with body weight and body fat [1]. Relatively few studies have explicitly manipulated fiber content of hypocaloric weight loss diets. Two Scandinavian studies found increased weight loss on a diet incorporating fiber supplements, as well as greater improvement in blood pressure, lower hunger ratings, and better dietary adherence [2, 3]. Thompson et al., using food-based sources of fiber, found no differences in weight loss between a high-fiber diet and other diets, but the weight losses reported for all groups (11-12% loss) were substantially higher than is typically seen in diet-only interventions [4]. A recent New Zealand study found that, over eight weeks, a high-protein diet produced greater weight loss than a high-fiber diet; however, both groups had exceptionally high fiber intake before and during treatment (≥25 g/day) [5].

Beans can contribute significant amounts of fiber to the diet, adding approximately 5–7 grams per half cup serving. Adult bean consumers have lower body weights, smaller waist circumferences, reduced systolic blood pressure, better overall nutrient intake, and greater intake of dietary fiber [6]. Beans have been shown to enhance satiety, delay the return of hunger, reduce the desire to eat something tasty, produce a smaller but longer-lived rise in plasma glucose, and greatly increase postprandial release of cholecystokinin, in some cases more than equal amounts of fiber from other sources [7–9].

The purpose of this preliminary study was to examine the nutritional effects and acceptability of incorporating higher amounts of dry beans in a weight loss diet over a four-week treatment period following a two-week baseline period. We compared two high-fiber hypocaloric diets differing in sources of fiber: (a) beans or (b) fruits, vegetables, and whole grains.

## 2. Materials and Methods

### 2.1. Subjects

Subjects were generally healthy men and women and were recruited primarily via broadcast messages in the Medical University of South Carolina e-mail system. Inclusion criteria included age from 18 to 70 and BMI = 27.0–35.0. Exclusion criteria included subject usage of medications affecting weight, history of diabetes, and pregnancy.

### 2.2. Measures

*Rating Scales*. Questionnaires elicited ratings of hunger, satiety, tolerability and acceptability of the diet, and likelihood of continuing the diet, using 6-point Likert scales. 

*Food Logs*. Subjects kept a food diary three days per week (2 weekdays, 1 weekend day). 

*Power of Food Scale (PFS) [10]*. The PFS is a 15-item self-report measure of hedonic hunger (i.e., food-related thoughts and desires unrelated to physiological need). 

*Eating Behavior Inventory (EBI) [11]*. The EBI is a 26-item measure of behaviors conducive to weight control, including both positive and maladaptive weight management behaviors; higher EBI total scores indicate a greater usage of weight control behaviors. EBI scores consistently improve following intensive behavioral weight loss interventions, and greater increases are associated with greater weight loss [12].

### 2.3. Diet Conditions

Both diets recommended a target caloric limit of 1400 kcal/day and at least 25 g of fiber daily. To achieve the fiber goals, the standard high fiber (SHF) diet emphasized whole grains, fruits, and vegetables, while the bean diet used 1.5 cups of dry beans/day. Subjects were counseled to gradually increase their fiber intake to the target level over the first week of the diet. Subjects received a cookbook of high-fiber meals and $20.00 per week of high-fiber foods, both appropriate to their diet, as well as BEANO, an enzyme-based dietary supplement that is used to reduce gas in the digestive tract, for any needed self-treatment.

### 2.4. Procedures

After a 2-week baseline period, subjects were randomly assigned to the SHF or bean diet for a 4-week treatment period. Subjects kept a 3-day food log in each of the six weeks. Rating scales were completed by subjects at each visit starting with the baseline visit. The PFS and EBI were completed at the screening visit and at the final (week 4) visit.

### 2.5. Data Analysis

Food log data on food and beverage intake were entered into Nutritionist Pro for nutrient analyses. For each nutritional variable, a daily average was calculated for each week from the data of three days for that week. Baseline period data were averaged across the two baseline weeks. Thus, a total of 5 data points were created for each nutrient variable.

Statistical Package for the Social Sciences (ver. 19) was used for statistical analyses. General linear model analyses with repeated measures were used to compare groups across time.

## 3. Results

20 healthy overweight and obese subjects (18 females, 2 males) were enrolled and randomized into this study. One female patient randomized to the SHF group withdrew the consent, so the resulting groups had *n* = 10 (Bean group) and *n* = 9 (SHF group). The mean age of participants was 46.9, and mean BMI was 30.6.

### 3.1. Weight Loss

Both groups lost weight over the four weeks (*P* < 0.001), with no significant difference between groups (SHF: 1.08 kg, SD = 1.70; Bean: 1.62 kg, SD = 1.26). All Bean group subjects lost weight while 3 of 9 members in SHF diet gained weight during the treatment period.

### 3.2. Dietary Fiber Intake

**Figure 1:**
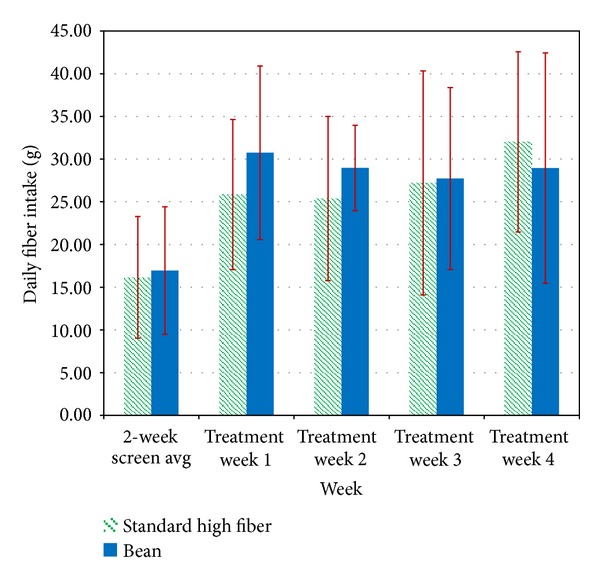
Daily fiber intake. Fiber intake increased significantly over time (*P* < 0.001) for both groups, with no difference between groups. The Bean group averaged 29.10 g/day (SD = 4.9) fiber over the treatment period (compared to 16.95 g/day (SD = 7.46) at baseline), and the SHF group averaged 28.85 g/day (SD = 8.2) (compared to 16.16 g/day (SD = 7.12) at baseline (Figure 1). Bean group members met the dietary fiber intake goal (25 g/day) M = 3.10 out of 4 weeks, and SHF group members met the goal M = 2.33 out of 4 weeks (*P* = 0.113).

Fiber intake increased significantly over time (*P* < 0.001) for both groups, with no difference between groups. The Bean group averaged 29.10 g/day (SD = 4.9) fiber over the treatment period (compared to 16.95 g/day (SD = 7.46) at baseline), and the SHF group averaged 28.85 g/day (SD = 8.2) (compared to 16.16 g/day (SD = 7.12) at baseline (see Figure 1). Bean group members met the dietary fiber intake goal (25 g/day) M = 3.10 out of 4 weeks, and SHF group members met the goal M = 2.33 out of 4 weeks (*P* = 0.113).

### 3.3. Caloric Intake

**Figure 2:**
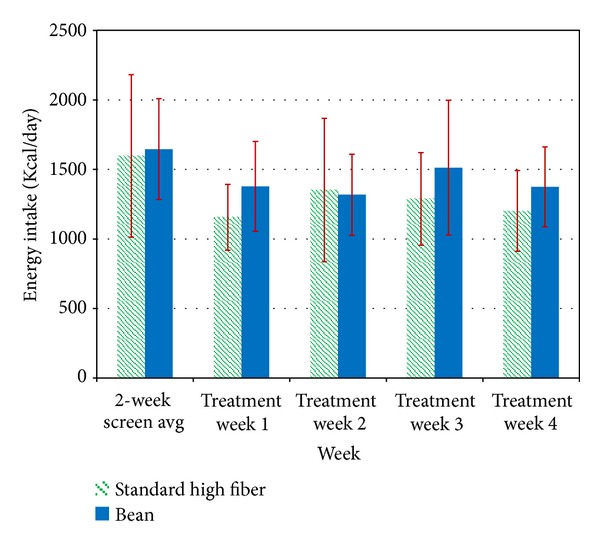
Daily caloric intake. There was a significant reduction in caloric intake (*P* = 0.004) for both groups, with no significant difference between groups. The Bean group averaged 1387 calories/day (SD = 252) over the treatment period (compared to 1646 kcal/day at baseline; SD = 363), and the SHF group averaged 1250 calories/day (SD = 297.0) over the treatment period (compared to 1597 kcal/day at baseline; SD = 584) (Figure 2). Bean group members met dietary caloric intake goals (1400 kcal/day) M = 2.20 out of 4 weeks, and SHF group members met the goals M = 2.67 out of 4 weeks (*P* = 0.44).

There was a significant reduction in caloric intake (*P* = 0.004) for both groups, with no significant difference between groups. The Bean group averaged 1387 calories/day (SD = 252) over the treatment period (compared to 1646 kcal/day at baseline; SD = 363), and the SHF group averaged 1250 calories/day (SD = 297.0) over the treatment period (compared to 1597 kcal/day at baseline; SD = 584) (see Figure 2). Bean group members met dietary caloric intake goals (1400 kcal/day) M = 2.20 out of 4 weeks, and SHF group members met the goals M = 2.67 out of 4 weeks (*P* = 0.44).

### 3.4. Energy Density

**Figure 3:**
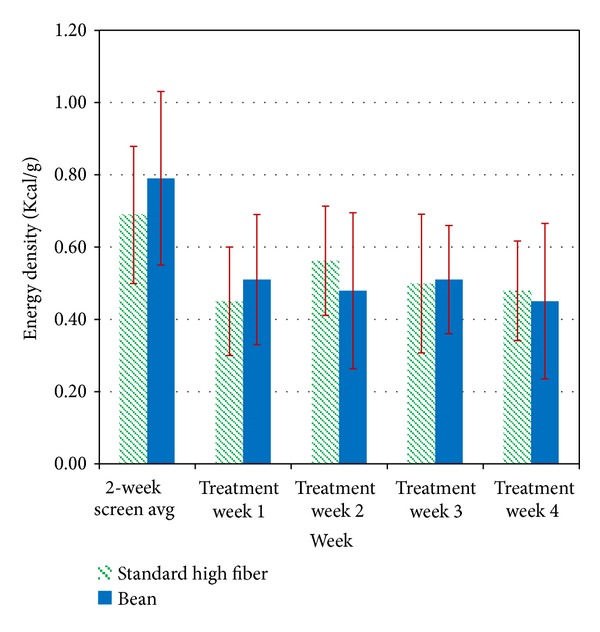
Energy density of daily caloric intake. Energy density (kcal/g) was calculated as total calorie intake/total weight of all recorded food and beverages including water. Both groups showed significant reductions from baseline in energy density throughout the 4-week treatment (*P* < 0.001). The Bean group showed a reduction from 0.79 Kcal/g (SD = 0.19) at screening to an average over treatment of 0.49 Kcal/g (SD = 0.01), while the SHF group showed a similar reduction from 0.69 Kcal/g (SD = 0.24) to 0.50 Kcal/g (SD = 0.15) (Figure 3). This was accomplished by increasing weight of intake while decreasing caloric intake. The Bean group showed a significantly greater increase in intake weight (M = 853 g; SD = 703) than did the SHF group (M = 210; SD = 318; *P* < 0.05).

Energy density (kcal/g) was calculated as total calorie intake/total weight of all recorded food and beverages including water. Both groups showed significant reductions from baseline in energy density throughout the 4-week treatment (*P* < 0.001). The Bean group showed a reduction from 0.79 Kcal/g (SD = 0.19) at screening to an average over treatment of 0.49 Kcal/g (SD = 0.01), while the SHF group showed a similar reduction from 0.69 Kcal/g (SD = 0.24) to 0.50 Kcal/g (SD = 0.15) (see Figure 3). This was accomplished by increasing weight of intake while decreasing caloric intake. The Bean group showed a significantly greater increase in intake weight (M = 853 g; SD = 703) than did the SHF group (M = 210; SD = 318; *P* < 0.05).

### 3.5. Power of Food Scale

Both groups exhibited a significant reduction in total PFS score over time (*P* = 0.002), indicating a reduction in hedonic hunger. The SHF group tended to exhibit a lower PFS at both assessments (*P* = 0.077), with no group differences in amount of reduction. The Bean group's PFS score was reduced from 2.47 (SD = 0.73) to 2.03 (SD = 0.47) and that of the SHF group from 2.05 (SD = 0.72) to 1.53 (SD = 0.48).

### 3.6. Eating Behavior Inventory

EBI scores for both groups increased significantly from baseline (M = 82.7, SD = 8.15) to posttreatment (M = 102.2, SD = 7.84; *P* < 0.001), indicating increased use of weight control behaviors. There was no significant interaction between treatment group and EBI change.

### 3.7. Rating Scales

For both groups, self-rated hunger was significantly reduced from baseline throughout the treatment period (*P* = 0.008), with no difference between treatment groups. Self-reported fullness increased similarly in a linear fashion for both groups over the treatment period (*P* = 0.003). Both diets were consistently rated highly on the question “in the past week, how much did you like the diet you were on?” with an average rating of 2.0 (SD = 1.13) on the 1–6 scale, with 1 representing the most favorable rating. At all time points, subjects in both groups reported minimal problems tolerating their diet because of physical discomfort (M = 5.3; SD = 0.92; 6 = “no problems at all”). The likelihood of following the diet for up to 6 months was rated as high by subjects in both groups (M = 2.0, SD = 1.09; 1 = extremely likely). There was a nonsignificant trend for the SHF diet to be rated as easier to follow (*P* = 0.080), with no changes in this rating over time.

## 4. Discussion

The purpose of this short-term pilot trial was to assess the feasibility and effects of a high-fiber weight loss diet focusing on dry beans as the primary source of fiber. Results indicate that this diet achieved levels of adherence and satisfaction similar to those seen with a diet relying on more commonly recommended sources of fiber. Both diet groups demonstrated a statistically and clinically significant increase in fiber intake above baseline intake, with average daily intake during treatment of approximately 29 grams in both groups and a 75% increase compared to baseline. The achieved level of fiber intake was within the range of recommended dietary guidelines and even greater than the level of fiber intake that each diet plan targeted (25 grams). This level is also much greater than that typically observed in weight loss diets, even those that explicitly promote increased fiber intake [13].

Similarly, both diets produced significant and comparable reductions in caloric intake. They also produced reductions in energy density, reflecting both a drop in caloric intake and an increase in the weight of intake, the latter of which was greater in the Bean group. Intakes lower in energy density have been shown to be related to greater weight losses [14].

Both groups reported a significant increase in fullness and reduction in hunger during the 4-week treatment period. It is noteworthy that subjects reported levels of hunger during the treatment period that were lower than reported at baseline, despite the fact that they were consuming approximately 250–350 calories per day less than during baseline. Further, despite the caloric reduction, rated levels of fullness increased over the treatment period. These findings suggest an important role for fiber in increasing satiety and therefore possibly improving adherence to a hypocaloric diet. This is consistent with earlier studies which found that weight loss diets including fiber supplement tablets produced better adherence and lower hunger relative to lower-fiber diets [2, 3].

Acceptability of both diets was high. Subjects rated their assigned diet quite favorably and reported being able to follow it for as long as six months. This might appear surprising, as substantial increases in dietary fiber, particularly from beans, are often assumed to produce gastrointestinal discomfort. However, subjects in both the Bean and Standard High Fiber groups reported only minimal problems of physically tolerating the diet. It should be pointed out that subjects were counseled to gradually increase their fiber intake to the target level over the first week of the diet and were provided BEANO for use as needed.

Limitations of this study include the short treatment period (4 weeks) and small sample size. It is not known how much the patients' compliance was enhanced by the provision of the recommended high-fiber foods, as well as by the required dietary self-monitoring and weekly study visits. However, this trial was not intended to be an efficacy or effectiveness trial. Rather, it was designed to assess the feasibility and acceptability of a high-fiber weight loss diet featuring dry beans, relative to an isocaloric high-fiber diet based on whole grains, fruits, and vegetables. This diet was found to have equal effects on nutritional intake and weight and to be equally acceptable and adoptable. Results support the value of high fiber content in weight loss diets and suggest that dry beans may be a viable source of fiber in such diets.

## 5. Conclusions

While being sufficiently hypocaloric to produce weight loss, both the standard high fiber diet and the bean diet significantly increased fiber intake by 75% (to recommended levels), increased satiation, and reduced hunger. The bean diet and the standard diet were rated similarly and favorably in terms of acceptability and tolerability. Results support increasing fiber in weight loss diets with a variety of fiber sources including dry beans, which offer substantial amounts of fiber per serving.

## Abbreviations

EBI: Eating behavior inventory
PFS: Power of food scale
SHF: Standard high fiber.

## References

[1] Slavin JL (2005). Dietary fiber and body weight. *Nutrition*.

[2] Ryttig KR, Tellness G, Haegh L, Boe E, Fagerthun H (1989). A dietary fibre supplement and weight maintenance after weight reduction: a randomized, double-blind, placebo-controlled long-term trial. *International Journal of Obesity*.

[3] Rigaud D, Ryttig KR, Angel LA, Apfelbaum M (1990). Overweight treated with energy restriction and a dietary fibre supplement: a 6-month randomized, double-blind, placebo-controlled trial. *International Journal of Obesity*.

[4] Thompson WG, Holdman NR, Janzow DJ, Slezuk JM, Morris KL, Zemel MB (2005). Effect of energy-reduced diets high in dairy products and fiber on weight loss in obese adults. *Obesity Research*.

[5] Te Morenga LA, Levers MT, Williams SM, Brown RC, Mann J (2011). Comparison of high protein and high fiber weight-loss diets in women with risk factors for the metabolic syndrome: a randomized trial. *Nutrition Journal*.

[6] Papanikolaou Y, Fulgoni VL (2008). Bean consumption is associated with greater nutrient intake, reduced systolic blood pressure, lower body weight, and a smaller waist circumference in adults: results from the National Health and Nutrition Examination Survey 1999–2002. *Journal of the American College of Nutrition*.

[7] Gell PB, Anderson JW (1994). Nutrition and health implications of dry beans: a review. *Journal of the American College of Nutrition*.

[8] Bourdon I, Olson B, Backus R, Richter BD, Davis PA, Schneeman BO (2001). Beans, as a source of dietary fiber, increase cholecystokinin and apolipoprotein B48 response to test meals in men. *Journal of Nutrition*.

[9] Leathwood P, Pollet P (1988). Effects of slow release carbohydrates in the form of bean flakes on the evolution of hunger and satiety in man. *Appetite*.

[10] Lowe MR, Butryn ML, Didie ER (2009). The Power of Food Scale. A new measure of the psychological influence of the food environment. *Appetite*.

[11] O’Neil PM, Currey HS, Hirsch AA (1979). Development and validation of the Eating Behavior Inventory. *Journal of Behavioral Assessment*.

[12] O’Neil PM, Rieder S (2005). Utility and validity of the Eating Behavior Inventory in clinical obesity research: a review of the literature. *Obesity Reviews*.

[13] Gardner CD, Kiazand A, Alhassan S (2007). Comparison of the Atkins, Zone, Ornish, and LEARN diets for change in weight and related risk factors among overweight premenopausal women: the A to Z weight loss study: a randomized trial. *The Journal of the American Medical Association*.

[14] O'Neil PM, Cronan GE, Turner TF (2010). Changes in dietary energy density with participation in a 12-week weight loss trial using a commercial format. *Obesity*.

